# “The Squeaky Wheel Gets the Grease”: Rental Assistance Applicants’ Quests for a Rationed and Scarce Resource

**DOI:** 10.1093/socpro/spab035

**Published:** 2021-08-17

**Authors:** Danya E. Keene, Alana Rosenberg, Penelope Schlesinger, Shannon Whittaker, Linda Niccolai, Kim M. Blankenship

**Affiliations:** 1 Yale School of Public Health,; 2 American University

**Keywords:** rental assistance, public housing, social services, criminal justice, qualitative

## Abstract

In 2016, only one in five eligible U.S. households received rental assistance and waiting lists averaged two years nationally. The gap between available rental assistance and need requires systems to allocate this scarce resource. The way potential rental assistance recipients experience and navigate these systems is likely to shape who ultimately receives assistance. We draw on repeated qualitative interviews (N=238) with low-income New Haven residents (N=54) to examine how participants understand and navigate rental assistance applications and waiting lists. Participants encountered multiple challenges in their search for rental assistance. They described an opaque and complex application and waiting process requiring significant knowledge to navigate. They also described considerable labor associated with monitoring waiting lists, a challenge made more difficult for some by their lack of a stable address. Additionally, participants described significant labor and knowledge required to strategically navigate prioritization systems that often required them to advocate for their deservingness of scarce housing resources. Our findings suggest that the allocation of rental assistance through complex processes that depend on applicant knowledge, labor, and advocacy may create barriers to housing, particularly for more vulnerable and marginalized housing seekers.

The U.S. Department of Housing and Urban Development (HUD) currently provides rental assistance to approximately five million U.S. households through tenant and project-based subsidies that reduce tenants’ rent to 30 percent of their income. These forms of rental assistance are important resources for low-income U.S. households. In an unaffordable housing market, they provide one of the few available sources of affordable and adequate housing (Joint Center for Housing Studies 2020). Indeed, research shows that individuals who receive rental assistance report more housing stability, fewer housing cost-burdens, and greater housing satisfaction than those on rental assistance waiting lists (Schapiro et al. 2021). By improving housing access, rental assistance may also contribute to the health and well-being of low-income Americans. An extensive body of research suggests that adequate, affordable, and stable housing is important to psychological and physical well-being and behavioral health (Benfer and Gold 2017; Hernández and Swope 2019). Furthermore, emerging research indicates health benefits of rental assistance for both adults and children (Fenelon et al. 2017; Keene et al. 2020; Simon et al. 2017; Slopen et al. 2018).

While rental assistance is a valuable resource, it is also in short supply. In 2016, only one in five eligible U.S. households received rental assistance, and waiting lists averaged two years nationally (Fischer and Sard 2017). The gap between available rental assistance and need requires systems to ration and distribute this scarce resource. The federal government (HUD) contributes to this system by setting broad eligibility criteria for rental assistance. However, access is primarily determined by local housing authorities who often develop their own eligibility criteria and rationing systems (Leopold 2012). Recent research has begun to examine these rationing systems by analyzing official housing authority policies (Curtis, Garlington, and Schottenfeld 2013; Zhang and Johnson 2018). Additionally, a small body of literature has examined the ways that these policies, which often involve considerable discretion, are implemented by front-line workers (Dickson-Gomez et al. 2007). However, largely absent from this literature is the perspective of those who need or seek rental assistance. The way potential recipients of this scarce resource perceive, experience, and navigate these policies and practices is likely to shape who ultimately receives rental assistance, and even who applies for it in the first place. Indeed, researchers have argued that understanding how people respond to and experience policies that are directed at them is critical to evaluating their efficacy, as well as their potential unintended consequences (Burris et al. 2004).

In this paper, we draw on repeated qualitative interviews (N=238) with low-income New Haven residents (N=54) to examine how participants understand and navigate subsidized housing applications and waiting lists. Our analysis contributes to an understanding of rental assistance policies and their consequences. More broadly, we use the example of rental assistance to better understand the bureaucratic rationing of social welfare resources from the perspective of potential recipients themselves.

## BACKGROUND AND PRIOR RESEARCH

### Rationing of Social Welfare Resources

Our study builds on an existing literature that has examined the allocation of social welfare resources through official “on the books” policies, as well as through the actions of frontline workers or “street-level bureaucrats” who often have considerable discretion in how they allocate resources (Lipsky 1984; Morgen 2001; Watkins-Hayes 2009). Within this larger literature, a smaller body of research has examined the experiences of those who apply to social welfare programs. In particular, it has examined how negative experiences with social welfare applications, including stigma, bureaucratic red tape, and long wait times, can deter applicants from seeking or receiving government resources (Barnes and Henly 2018; Edin and Shaefer 2015; Soss 1999).

In their pursuit of social welfare resources, individuals must navigate complex state bureaucracies, and this process is associated with a range of challenges, often referred to in the literature as “administrative burdens” (Moynihan, Herd, and Harvey 2015). First, those seeking social welfare resources face the burden of “learning costs”: they must obtain knowledge about eligibility, the nature of services, and how to access them. Resource seekers may also contend with “psychological costs” in the form of stigma, stress, or a loss of autonomy associated with program participation. Finally, the completion of applications, reenrollment, and ongoing documentation comprise “compliance costs.”

Existing literature suggests that these administrative burdens and bureaucratic hurdles can reduce participation in social welfare programs (Moynihan et al. 2015). For example, means-tested programs, that require applicants to prove income eligibility, have much lower take-up rates than universal programs like Social Security and Medicare (Moynihan et al. 2015). Other research finds that simplifying application processes and reducing burdens can increase program take-up (Hanratty 2006; Klerman and Danielson 2011). In addition to reducing overall take-up of social welfare resources, some evidence suggests that administrative burdens can have differential impacts, depending on applicants’ ability and resources to overcome them. For example, Herd (2015) finds that administrative burdens contribute to lower take-up of assistance from the Supplemental Nutrition and Assistance Program (SNAP) among older adults relative to other eligible age groups who may be better able to navigate the process. In their study of how administrative burdens affect access to TANF benefits, Brodkin and Majmundar (2010) find that administrative burdens had a larger impact on program retention for those who did not have high school degrees, never married, or experienced deep poverty. As Moynihan (2015) and colleagues note, stressors associated with poverty may make administrative burdens particularly difficult to overcome and thus may restrict access to resources for those who need them most.

Ultimately, cumbersome and bureaucratically complex application processes may help ensure that resources are reserved for intended recipients, but they may also deter eligible applicants and restrict access. In this sense, some scholars have argued that administrative burdens are a hidden and politically more attractive way to reduce public spending (Hacker 2004; Lipsky 1984). According to Lipsky (1984:9), these burdens contribute to “bureaucratic disentitlement,” which “works to deter those seeking aid by signaling that its receipt depends on luck, connection, persistence, or other factors over which people have little control.”

Some existing qualitative work has examined the ways that administrative burdens or application challenges are experienced and interpreted by potential clients of social welfare programs (Barnes and Henly 2018; Soss 1999). Our analysis builds on this literature to consider the experiences of those seeking rental assistance. Federal rental assistance is different from other social welfare programs because it is available only to a small portion of eligible applicants. Thus, housing authorities must determine not only eligibility, but also how to prioritize who will receive assistance from such a scarce resource. In this process of prioritization, program administrators often must make decisions about who needs housing most or who is more deserving of this resource, thereby reflecting and perhaps reinforcing societal values. Furthermore, the scarcity of rental assistance contributes to low probabilities of success, which may combine with other application challenges to deter some applicants. Additionally, the uncertainty of success associated with rental assistance applications and the labor associated with this process may contribute to stress, a form of administrative burden that has been understudied in the existing literature (Moynihan et al. 2015).

### The Allocation of Federal Rental Assistance

The U.S. Department of Housing and Urban Development (HUD) provides rental assistance to approximately five million families in two primary forms: project-based assistance and tenant-based assistance. Project-based housing includes public housing developments that are operated by local housing authorities, as well as privately owned affordable units and developments that are subsidized through grants to their owners. Tenant-based housing is provided in the form of Housing Choice Vouchers (HCVs), formerly called Section 8 Vouchers, which are issued to tenants to subsidize private market rent. These rental assistance programs, available to families earning below 50 percent of the area median income, make housing affordable by setting a tenant’s rent at 30 percent of their income. Given that market-rate rent is out of reach for low-income earners in most areas of the country, rental assistance is a critical source of affordable housing. In fact, in many parts of the country, there are zero available, affordable, and adequate *unassisted* rental units for low-income earners (McDonald and Poethig 2014). Despite its value, only one in five eligible households currently receive rental assistance, and waiting time on the lists average 26 months nationally (Fischer and Sard 2017).

The scarcity of rental assistance necessitates policies to ration it. In 1979, Congress set federal guidelines for the allocation of HUD rental assistance, granting priority to those with severe rent burdens and those who had been displaced by government programs (Leopold 2012). However, these preferences were removed in 1998 with the Quality Housing and Work Responsibility Act that granted more discretion to local housing authorities in determining the allocation of housing resources. Today, HUD sets an income threshold for rental assistance recipients and requires housing authorities to ensure that 75 percent of new voucher recipients and 40 percent of new public housing residents have extremely low incomes (less than 30 percent of AMI) (Leopold 2012). However, beyond these broad requirements, local housing authorities have tremendous discretion in allocating rental assistance. This discretion has resulted in considerable variability in admission processes across housing authorities.

As one example, local housing authorities vary widely in considering alcohol, drug and criminal histories in their determination of eligibility (Curtis et al. 2013; Purtle et al. 2020). Rental assistance may be particularly valuable to individuals with recent criminal justice or incarceration histories, given the many barriers to securing private market housing that they face, including stigma and background checks (Geller and Curtis 2011). However, criminal justice histories can also create unique eligibility barriers to receiving assistance. HUD imposes a mandatory three-year ban on individuals who were evicted from public housing for drug or criminal involvement; however, local public housing authorities can set additional restrictions. In some housing authorities, even having a history of arrest can be grounds for exclusion from rental assistance, while in other places, only felony convictions are taken into account. Housing authorities also vary in the length of the look-back periods that they consider in evaluating an applicant’s record and the process of negotiating or appealing eligibility denials (Purtle et al. 2020). In addition to this variation in official policy, existing policies often grant considerable discretion to local workers in determining eligibility decisions (Curtis et al. 2013). Our own prior qualitative work, conducted among individuals with recent incarceration histories, finds that navigating this discretion and uncertain eligibility can create additional barriers to rental assistance, as applicants must work to prove themselves deserving of this scarce resource in a landscape where they encounter considerable stigmatization associated with their criminal justice histories (Keene et al. 2018).

Not only do housing authorities vary in whom they consider eligible for admission, they also vary in how they govern their waitlists. Approximately one quarter of housing authorities allocate housing on a first-come, first-served basis, and another quarter employ some form of lottery (Leopold 2012). The remainder employ tiered waitlists to prioritize certain categories of applicants (Leopold 2012). For example, many housing authorities prioritize local applicants, those with disabilities, and those with dependent children. Indeed, a significant portion of the country’s public housing developments are reserved for individuals with disabilities or older adults. Additionally, approximately one quarter of housing authorities have some preferential admission for homeless individuals who are thought to have the most immediate housing needs (Dunton et al. 2014). In addition to traditional rental assistance programs (public housing and vouchers), some HUD funded housing is available for individuals with particular health needs. For example, HUD provides housing to individuals living with HIV through a range of programs.

These tiered waiting lists and population specific housing programs often reflect values about who needs and who deserves housing (Zhang and Johnson 2018). These systems of prioritization are also likely to affect the experience of applicants themselves. For example, applicants’ efforts to position themselves favorably within tiered waiting lists may be an added form of labor or “administrative burden” that contributes to stress or places those with fewer resources at a disadvantage. In general, the complexity of the rental assistance application process and its varied landscape across housing authorities may contribute to opacity and confusion that affects applicants’ experiences and, ultimately, their success in obtaining housing (Moore 2016).

Though prior research has explored the various processes that housing authorities employ to allocate rental assistance (Leopold 2012; Moore 2016), we currently know little about how applicants or potential applicants understand and experience these systems. Understanding these experiences is critical to examining the impact of these policies, because perceptions and experiences of the system are likely to influence how applicants navigate the application process. These perceptions are also likely to shape who applies for housing in the first place and may help explain low rates of application among eligible populations that have been documented in the literature (Moore 2016). Our study responds to this gap by capturing the perspective of both housing applicants and non-applicants through qualitative interviews.

## METHODS

### Overview

This study takes place in New Haven, Connecticut, a city with approximately 130,000 residents. Although small, New Haven experiences many of the challenges that larger cities face, including high rates of poverty, shortages of affordable housing, and long waiting lists for rental assistance (Rawlings 2013). In 2013, rent-assisted households in New Haven County spent an average of 23 months on public housing waiting lists and 25 months on waiting lists for vouchers (HUD 2013). As is the case in many cities (Leopold 2012), the Housing Authority of New Haven has a tiered application process for rental assistance that offers preference to certain applicants, rather than administering aid on a purely first-come, first-served basis (Elm City Communities 2021).

Our analysis draws on qualitative data collected as part of a longitudinal cohort study of 400 low-income residents of New Haven, CT. The Justice, Housing and Health Study (JustHouHS) was designed to examine the intersections of the criminal justice system, housing, and health, with a particular focus on sexual health and HIV risk. Low-income New Haven residents (defined for this project as those who were homeless, received food assistance or Medicaid, or resided in census tracts with greater than 20 percent poverty) were recruited using a combination of flyers posted throughout the New Haven community, outreach from service providers, and snowball sampling. Given the study’s interest in the intersection of mass incarceration and health, the sample was stratified to include 200 individuals released from prison or jail in the last year. Rental assistance was a significant focus of the JustHouHS study and all participants were income-eligible for this resource. However, participants were not recruited with respect to rental assistance status and thus had a range of experiences with rental assistance programs. This variation allowed us to examine participant experiences at different stages of the application process.

JustHouHS participants took a baseline survey in the fall of 2017 and returned to take a follow-up survey every six months for a total of five surveys. A subset of JustHouHS participants (N=54) also completed qualitative interviews every six months. We conducted five rounds of interviews over three years, with the final round occurring virtually in May of 2020. Fifty-one (94 percent) participants completed more than one interview. Forty-three (80 percent) completed all four in-person interviews and 39 (72 percent) participated in the May 2020 virtual interviews. At each wave, four to five (7–9 percent) participants were not able to participate due to incarceration. Our analysis draws on the 238 total interviews that participants completed across study waves.

### Sample

Qualitative participants were selected purposively from the survey sample to maximize variation with respect to criminal justice history and race. Among the 54 participants, 37 were male and 17 were female. While rental assistance recipients are predominantly female (HUD), our largely male sample likely reflects the study’s over-sampling of individuals who were recently released from prison. Thirty-four participants identified as African American, nine as white, and eleven as other. Ten identified as Latinx. Participants’ average age was 45. Consistent with the study’s sampling framework, 27 had a recent (within one year) history of incarceration at baseline. At baseline, 17 reported being homeless during the last six months. With respect to rental assistance, 12 participants were receiving rental assistance at baseline. An additional 24 participants applied for rental assistance at some point during or prior to the study. Among these, seven obtained assistance by the last interview. The remaining 18 participants had neither applied for nor obtained rental assistance. Some of these 18 participants discussed barriers to rental assistance applications.

### Data Collection

A team of researchers (first, second, and third authors) conducted interviews between November 2017 and June 2020. Interviews lasted between 13 and 155 minutes, with an average time of 54 minutes. All interviews except the last wave took place in a private room at the study office and were audio recorded and transcribed by a professional transcriptionist. The last interview was conducted virtually, audio recorded, and transcribed. The same interviewer conducted interviews with each participant across the study waves. Interview topics consisted of current and prior housing, criminal justice involvement at both the individual and community level, economic situation, sexual relationships, condom use, HIV testing, health, relationships with family, friends, and partners, family background, and community connections.

### Analysis

Our analysis relied on a multi-stage coding process as described by Deterding and Waters (2021). We first employed broad codes to sort data into topical categories (e.g., relationships, employment, social networks, housing, housing assistance, health) using NVivo software. We then extracted and reviewed all excerpts associated with the index code “housing assistance” and utilized an open coding and memo-writing process to develop concepts related to participants’ search for rental assistance. All authors discussed and iteratively refined our list of open codes at a series of meetings. Next, we applied this list of codes to a portion (approximately one third) of the data that had been indexed under the housing assistance code using NVivo software. We then reviewed these excerpts and refined our codes into the broader themes that are described in each subsection of our results. We then reviewed all of the data indexed as housing assistance and organized them according to these themes. We also reviewed full transcripts to clarify and contextualize certain excerpts. During this process, we refined themes and subthemes based on emerging insights from our review of the coded data.

In addition to our coding, our analysis relied on longitudinal matrices that we created to describe participants’ housing and other project specific domains across time. These matrices were one-page charts containing brief summaries of the following housing topics for each interview wave: description of current housing situation, evaluation of current housing situation, and housing goals. We used these matrices to identify key transcripts to review in-depth and to provide context for coded excerpts. In presenting our findings below, we use pseudonyms, which were selected by participants themselves.

## FINDINGS

Participants discussed multiple challenges in their search for rental assistance. They described an opaque and complex application and waiting process that required significant knowledge to navigate. The opacity of the application process was exacerbated by differing rules across multiple housing authorities and by multiple gatekeepers, including individual landlords. Individuals with criminal justice histories encountered an additional layer of uncertainty due to complex and often unclear eligibility restrictions. Participants also discussed significant labor required to navigate the application process. First, they described the need to monitor waiting lists, a challenge made more difficult for some by their lack of a stable address. Participants also described significant labor and advocacy required to strategically navigate prioritization systems. They not only needed to prove their own housing needs, they also described the importance of engaging outside advocates to assist in this process. While advocates, prioritization systems, and targeted housing resources accelerated housing access for some, for others these systems seemed to create barriers. Nevertheless, many participants seemed to perceive the labor associated with rental assistance applications to be a worthwhile investment, given the potential value of this resource. As one participant, Mark, noted, “I know so many people who have gotten housing … and it just changes your life around.”

### An Uncertain Waiting Process

One challenge that participants described was lack of clarity about the application and waiting process. Some participants were unsure how to determine whether waiting lists were open and how to access them. As Liz noted, “I’m waiting for all the openings to open up. Nothing’s open right now. ‘Cause there was a thing going around saying New Haven was open. I went down there and they told me they were closed.”

Participants were also uncertain about how the lists worked and what factors determined their own movement on them. Several participants had been on waiting lists for a long time and were uncertain how much longer they would need to wait. As Brooklyn noted, “They say probably, like, another year to two years, so, yeah, I know, sucks.” Neveah expressed frustration at the length of the wait, noting, “Section 8? I have applied for it. I don’t understand why I haven’t gotten it yet, but, yeah, I definitely applied for it.”

Others described updates from the housing authority as unclear. For example, Tyler had been waiting on housing authority lists for over two years and was desperate to leave the shared transitional housing room where he had been staying. He finally received a letter from the housing authority asking whether he wanted to remain on the waiting list, but he wasn’t sure how to interpret it, noting, “But some people are telling me that that means that they’re getting ready to house me.”

Some participants also described the waiting lists’ movement as illogical. As Mark explained, “the city housing down there I’m on the waiting list, which is a – it’s crazy because I went and signed up as a joke – you’re 961. So I went back and checked again. Oh, you’re 1087. I was like, “How did I go up?” Indeed, in many locations, rental assistance is not allocated on a purely first-come, first-served basis, but rather is based on a range of factors such as the perceived urgency of an applicant’s need and fit between household characteristics and available units. For some participants, this complex system of prioritization contributed to the lack of transparency about the process and a lack of trust in the system.

Some participants were deterred by the length and nature of the wait. As Mark noted above, he signed up for rental assistance as “a joke” suggesting that he did not consider obtaining it as a realistic possibility. Several participants noted that you needed to “be lucky” to ultimately obtain rental assistance, and, as a result, they needed to figure out how to survive without it. As Cora noted, “I don’t depend on it … but I have faith that maybe one day I could be a lucky one that gets low income housing.” Liz explained that she needed a place to live today and couldn’t afford to wait for the uncertain possibility of receiving assistance. After many months of waiting for the rental assistance lists to open, she began looking into other options.

In other situations, the uncertainty about how the lists moved could catch participants by surprise when they reached the top. For example, Trinity was told that a public housing unit was available after she had already paid that month’s rent at her current apartment. Though she was excited to finally receive rental assistance, she described the challenge of coming up with a security deposit for her new apartment. She explained, “And the only thing I don’t like about it is that I had already paid my rent where I was at and they called me a week after that. And they told me if I don’t have the security [deposit], that I lose it and go to the bottom of the list.”

### Additional Uncertainty for Those with Criminal Justice Histories

Participants who had criminal records described an additional layer of uncertainty related to their eligibility. As Mark noted, “I don’t know how that works for – with the city. I don’t know if that will, um, affect it at all … Because I know they-they-they give like housing to people who I guess on probation and stuff.” Multiple participants were unsure if their particular criminal justice related circumstances would affect their eligibility, and some were not able to determine this until late in the application and waiting process. For example, Liz reached the top of the list only to be denied because there was a warrant on her record that she was not aware of. By the time she cleared the warrant she had lost her spot.

Jordan was also denied housing after a long wait. When he came for his third interview, he reported with excitement that he had been offered a public housing unit in a neighboring town. He had received a letter saying that he had reached the top of the list and could come interview for an apartment. However, at the next study interview, he reported that he had been denied the apartment due to his criminal record. He reported, “My criminal record. She said that she feels that I would bring drugs into the apartment and stuff, ‘cause you know, all my past is drug-related charges—but I haven’t had no drug charges in 15 years, so why are they gonna hold that against me?”

Similarly, Brandon described being denied housing due to his criminal record after spending many months on a neighboring town’s housing authority waiting list. He explained, “we waited all these months for me to get number one on the list and then you deny me. … Why you wait all these months, get my hopes up and then when I come here you deny me? Should have did that from the jump.” Brandon, Jordan, and Liz’s experiences of waiting for long periods of time only to be denied illustrate how the length of the waiting list can intersect with uncertain eligibility to create additional burdens for some rental assistance applicants.

While Brandon continued to apply for other sources of rental assistance and was ultimately successful, some participants were deterred by uncertain eligibility requirements and chose not to apply at all. For example, when asked if he had applied for housing assistance, Elijah replied, “I would but I’m not sure if … if, uh, I can get assistance. … I don’t think Housing Authority accept criminals.”

### Varying Rules and Multiple Gatekeepers Contribute to Uncertainty

The uncertainty of the application and waiting process seemed to be exacerbated by varying rules across multiple gatekeepers. Several participants had applied to multiple housing authorities to increase their chances of success. As Jordan noted, “Well, I got a letter from the Middletown Housing Authority too because I had put in applications in Middletown, Bristol, Wallingford and New Haven. So I got a lot of options out there.” These multiple housing authorities had differing rules, timelines, and procedures for managing their waiting lists. As Maya explained, “You have to go to each city and apply and the waiting list for New Haven is five years long. The waiting list for West Haven is closed. The one for Hamden, it’s two years long.”

These multiple gatekeepers posed particular challenges to those with criminal records, given variation in eligibility across housing authorities, programs, or privately managed buildings. For voucher recipients, the need to pass landlords’ screening processes could further exacerbate uncertainty surrounding criminal justice related eligibility. Multiple participants successfully met the housing authority’s definition of eligibility and received a voucher, but then encountered difficulty in finding landlords who would rent to them because of their criminal records. For example, in recounting the challenges that she faced finding an apartment to rent with her voucher, Cora explained,

The director from Section 8 told me herself that the only two people that are denied no matter what are sexual offenders and arsonists. … Okay, that’s fine. I don’t have either of those, either of those so, um – but the housing, uh, locations that they were sending me to were having a problem with my criminal background, quote/unquote. This is what they told me. “So we can’t accept you. Your credit” – they said, “We could always work around that but criminal history we don’t want those type of tenants in our building.”

Carter alluded to a similar challenge. When asked about the process of finding a place to use his new rental assistance (RAP) voucher, he explained, “I just need to find an apartment that really don’t like … do, like, background checks.” Though he was ultimately able to find a landlord who would overlook his criminal justice history, he had to settle for what he described as unsafe living conditions.

In contrast, Lee described receiving a RAP voucher (state rental assistance) and not being able to use it to secure housing. He explained, “Well you know, I’m pushing it now. Because I had a voucher which I’m in possession of and received of they sent me almost two years ago. But you know, I’m having – I don’t know if it’s racial profiling or what. And I’m pushing it now for them to investigate this. Because I should have been in a place, because I’m disabled. And they have not, you know, every time I called [caseworker], she said, ‘oh, it’s nothing yet.’ And this has been going on for a year and a half now.” In the end, Lee was not able to use his voucher. He rented an unsubsidized apartment that he stayed in until he was incarcerated 18 months into the study.

### A Labor-Intensive Waiting Process: Monitoring Lists

Participants also described the search for rental assistance as a labor-intensive process. Many described monitoring when lists were open to new applicants and the need to complete an application during these often short windows. Rather than passively waiting on lists, many participants actively and frequently monitored their status. Liz described calling the housing authority every two weeks to determine her spot on the list. Others visited the housing authority in person to check on their waitlist number. As Mark noted, “Every now and then I’ll go down there and find out where I am on the list. … And I haven’t been down there recently. But I will go down there and find out, like, where am I?” The study office was located near the housing authority’s central building and participants sometimes discussed plans to stop by after their interview or survey.

Among those who ultimately received rental assistance, a few participants attributed their success, at least in part, to this persistence. For example, Trinity noted, “I called, I stayed on top of them. And then near the end they called me.” Brandon, who obtained assistance in the final wave of the study, after years on the waiting list and multiple denials, noted that his persistent checking led him to be in the office of a particular facility on the day that an apartment became available. He explained, “I just came in to check on it and she had an apartment available. … And she showed me the apartment. I said, ‘I’ll take it.’ And that’s what happened. I came in that day and just so happened, there was an apartment available.”

Similarly, Leah, a mother of two young children with a recent incarceration history, obtained a voucher in the final wave of the study through a domestic violence program and attributed this success to her responsiveness and effort. She explained, “They got granted 20 Section 8 vouchers that they can give out to families, and if the families had their packets complete they would send them in. I was one of the first families that had my packet complete, so I got – it got sent in and I was the first round that got granted the voucher.”

Some participants described the application process and the need to monitor their status as burdensome. For example, when asked about her position on the waiting lists, Neveah replied, “I have no idea. I-I-I work so much it’s just like, you know, I don’t have time to chase.” Maya explained how the search for rental assistance required taking time off from work. She explained, “So I signed up for the lottery in Bristol. I haven’t heard anything yet. I’m going to go today— today I took a day off, number one, ‘cause I had to see you; number two, ‘cause I have to do more apartment searching; and number three, ‘cause I want to go to the housing authorities and see if I can put an application in or if I can apply for Section 8.”

Furthermore, the process of monitoring could be extra challenging for individuals who did not have an address of their own. For example, Malik was unsure if any updates about his status were reaching him because he had moved around from place to place. He explained, “I put my name on a list up here and I haven’t heard anything. I don’t know if my mail is still going—because at the time when I filled out for everything, I was still living on [H] Street.”

Cora, who was homeless, had recently begun to use her daughter’s house as a mailing address and explained that she needed to give this information to the housing authority so that she could receive notifications from them. She explained that notifications about an available unit required immediate response (within two weeks), and that failure to respond could result in losing her spot on the list after several years of waiting. She noted, “I have to go down there with my ID and just let them know a new address so they could change it right away, ‘cause as soon as they get that letter they’re gonna say show up at this time and this place and I have to make that.. … If not, a 2 could turn right back to 1,000 or they’ll just kick me off the list period and then I’ll be only waiting—I’d say maybe 2030.”

Similarly, Brandon, who was also homeless, relied on his sister’s address and noted the importance of responding to any updates about his status on the waiting lists. He explained, “They said the waiting list is a year to two. But they will be writing me within—throughout the year. And they looking for me to respond because if I don’t respond I get off the list. So all my mail goes to my sister house. That’s a definite. I can always use her address. Send it there and I get it. So I look forward to getting that every—every day I go there look for the mail. See if something in there for me.”

Brandon and Cora could receive mail at family members’ houses and were also able to proactively monitor their application status. However, some participants lost track of housing applications due to their housing instability. For example, Logan, explained, “They had us apply for housing authority. I got some letters for housing authority at Derby and I kind of lost contact, being homeless, then I got arrested. You know.” Logan lost his spot on the waiting list and did not reapply during the course of the study.

### A Labor-Intensive Process: Strategically Navigating Systems of Prioritization

Some participants not only monitored their status on waiting lists, but also worked to strategically position themselves for rental assistance. They recognized that rental assistance was not allocated on a first-come, first-served basis and that there were often spots reserved for individuals with particular needs. Indeed, most of the participants in our study who received rental assistance did so through targeted programs that allocated housing to those who were older adults, chronically homeless, had unique health needs, or, in one case, through a domestic violence prevention program.

Several participants noted the prioritization of chronically homeless individuals—those who were homeless for a consecutive year or experienced four or more episodes of homelessness in three years. Some worked to ensure that they were classified as such in the waiting process. For example, Mark emphasized his homeless status and dire need for housing in conversations with caseworkers at the addiction and recovery program that he attended. He explained, “‘Oh, well, where are you sleeping?’ I’ll say, ‘I’m gonna go sleep in my car.’ ‘Oh, you’re—you’re the number one priority. We gotta get you out of that car.’ You know?”

In a later interview, Mark explained that he chose to remain in a shelter despite the possibility of renting a room with his disability income because he did not want to risk losing his status as chronically homeless and thus his chances of receiving a voucher that would provide him with long-term affordability. He explained, “If I do that [rent a room] then it might kill the, um, kill down there [voucher application process]. I’m not sure—it would. It would, actually, stop that whole thing down the—where we’re going.”

Like Mark, other participants described shelters as important routes to housing assistance. Not only could these programs certify participants’ status as chronically homeless, they sometimes had vouchers that were reserved for their residents. Forexample, after many months of waiting on multiple housing lists, Maya described a plan to move out of her aunt’s apartment into a transitional housing shelter as a strategy to access housing that was allocated through this shelter. She explained, “Someone else told me if I go to [name of shelter], they have a program that helps people get Section 8 fast. You know? I don’t know.” Similarly, Luke explained, “Yeah, but you gotta go—you have to go through [the shelter] and you gotta stay there three to—one to three to four months before they—before you can, uh, get an apartment.” Though Luke preferred not to stay in this particular shelter, he viewed it as one of the only pathways to receiving rental assistance.

Beyond homelessness, some participants leveraged health needs as a strategy to obtain priority for rental assistance. Indeed, some rent-assisted units are reserved for individuals who have disabilities, and some participants noted that qualifying for disability was an important pathway to obtaining rental assistance. For example, Maya advised her husband to apply for disability benefits in an effort to secure housing for both of them after she completed a drug treatment program. She recounted:

Yeah. So I-I told him, “Let me do the program. While I’m in the program, you get your shit together with disability, because you’ll be able to get, you know, low-income housing”—there’s a lot of benefits that come with that. Even though it’s sad that you’re—you know, the way you gotta get it, I mean you’re sick, legitimately sick, you know, and like COPD [chronic obstructive pulmonary disease] is a serious thing, you know?

Josiah also considered signing up for disability in order to obtain housing, noting, “Um, uh, what I’ve seen is most of it [rental assistance], uh, have to do with disability, so I might sign up for that and that—I don’t know, maybe sign up for disability. Then if I get a job, cancel it or something. I don’t know.”

When Brandon tried to obtain rental assistance through a mental health program, like Josiah, he suggested that he did not necessarily need the services that the program provided, but rather considered it a route to getting housing. He explained, “One time I did ‘cause I tried to get into this mental health housing. They got programs for housing but you gotta be mental health evaluated. Alright. They sit there and talk to me and say, ‘Oh, I know what’s wrong with you, you schizophrenic. … Oh, Lord. Where’d you get that?’” Brandon later explained that he abandoned the strategy when the program wanted him to take medication that he did not think he needed. For some participants, the idea that housing was prioritized for those with disabilities served to discourage them from hopes of receiving assistance. For example, Miles explained, “Well, I applied for it [housing]. But to get housing assistance, you have to be, um, disabled some way. SSI or disability, they give it to you.”

In general, participants noted that it was difficult to obtain priority for rental assistance until they hit a point of desperation with either their health or their housing. For example, Kaylee explained that it was only after the third time that her family became homeless and after her husband attempted suicide that her case manager was able to successfully advocate for her to obtain a rent-assisted unit. As Maya noted above, in reference to her husband’s disability application, it was “sad” that he had to be so seriously ill to qualify for this resource.

### A Labor-Intensive Process: The Need for Advocacy

In their efforts to obtain priority for scarce housing resources, participants also described the importance of purposeful and often effortful self-advocacy. For example, when Sammy faced significant challenges with his current subsidized housing and sought to move to a new location, he wrote a letter advocating for his mental health needs. As he explained, “I started realizing, you know, this is—I am this close to collapse, you know? So I wrote a letter [laughs], a grievance letter to the director of [mental health service organization] saying, you know, this is—I’m in a very high-risk situation here, guys. … I went from number seven on the list to number one on the list over the weekend.” Several participants, like Sammy, noted the importance of speaking up for their needs when navigating the waitlist process. As Isaiah noted in reference to his rental assistance application, “The squeaky wheel gets the grease.”

Participants also drew on community members who could advocate on their behalf, including case managers, mental health counselors, medical providers, and even the staff of this study. For example, Isaiah discussed his plans to advocate for his housing needs in an upcoming appointment with his case manager explaining, “so I’m gonna have to go to her and connect and say, ‘Look, you know, what can be done at this point in time? You know, I’ve been homeless for over a year and, um, I don’t think I can do it for another year, so can you see what you can do on my behalf?’” In a subsequent interview, Isaiah was able to obtain a voucher with the support of his case worker, who also helped him find an apartment that he remained in for the remainder of the study.

Tyler also described the importance of having access to individuals who could advocate on his behalf. After waiting many months with no movement on the waiting lists, he asked his case worker at a local health center to write to the housing authority. He explained, “What—what I’m waiting on is … she’s [case manager] going to write a letter asking them why it took so long for me to get a place. Why is it taking so long that I—why am I on the list that—that far on the list when I’ve been on there since 2012. You know?”

Kaylee attributed her receipt of rental assistance early in the study, in part, to having access to advocates. She explained, “guess they have some sort of a meeting where all these different resources come together and the person that is handling or that we got the, um, voucher from was at the meeting, and she came with the voucher, and so my case manager was like, ‘That voucher is for *Kaylee* and her family.’”

In their interactions with both advocates and housing gatekeepers, participants also discussed a need to present themselves as deserving and appropriate candidates for housing. For example, when Cora appealed a denial of her application to a subsidized housing complex, she took care to manage her image. She explained, “So I said, fine, I’ll appeal it. And the big man, CEO of this housing complex, he came down … I dressed-dressed nice. I was showing him, you know, um, my resume, whatever, things. I am a working person.” Michael, who was participating in a drug treatment program, similarly noted that it was important to prove one’s competence in order to obtain one of the program’s housing vouchers. He explained, “Well, the 10 people who are doing the best with the program and working and doing, you know, all the legwork and all this stuff, they said they’ll give them people the vouchers for Section 8.” In their efforts to present themselves as worthy and in need of housing, participants suggested the importance of careful self-presentation. On the one hand, they needed to present their situation as dire. And on the other hand, they needed to show their competence as employed or employable adults.

## DISCUSSION

Participants described the search for rental assistance as a labor intensive and opaque process with uncertain returns on their investment of time and resources. While many described considerable expertise, skill, and agency in navigating this complex system, they also identified numerous challenges, including uncertainty about the application and waiting process, their eligibility to receive housing resources, and their ability to utilize housing vouchers once they received them. They also discussed the considerable labor associated with monitoring housing lists and strategically advocating for their housing needs in a system that was not first come, first served. The labor that participants invested in their quests for rental assistance suggests that they perceived great value in this resource.

Our data also suggest that the challenges and labor associated with the application process were not experienced equally. In particular, individuals who were homeless experienced additional difficulty in monitoring the waiting lists, due to their lack of stable address and the added day-to-day challenges of homelessness. Furthermore, echoing prior research, individuals with criminal justice histories faced additional uncertainties related to their eligibility and additional challenges navigating multiple gatekeepers (Keene et al. 2018). A few participants suggested that these criminal justice related barriers served to deter them from applying for rental assistance at all. The unequal challenges presented by administrative burdens suggest that reducing administrative barriers may improve access to rental assistance for the most disadvantaged housing seekers. Indeed, a recent review of housing authority policies and practices concluded that simplifying the application process and reducing the need for monitoring one’s waitlist status are important steps toward serving homeless applicants (Dunton et al. 2014).

Our data can also shed light on how participants experienced and responded to systems of prioritization that are designed to allocate scarce rental assistance resources. On the one hand, some participants were able to successfully leverage their unique needs to obtain housing. For example, multiple participants were prioritized for rental assistance due to their being chronically homeless individuals or having particular health challenges. On the other hand, the data suggest that the process of leveraging these needs required effortful advocacy, and, in many cases, access to individuals who could advocate on participants’ behalf and certify their housing needs as legitimate. In our study, not all participants with similar unique needs were able to successfully navigate this process. Indeed, nearly a third of our participants remained on housing authority waiting lists throughout the study, despite, in almost all cases, extreme housing needs and health issues.

Our data also suggest that participants’ efforts to position themselves for housing within this hierarchy may have unintended consequences. For example, a few participants reported staying in shelters rather than accepting temporary arrangements with friends or family members in order to qualify for priority housing. These efforts to ensure their status as homeless may adversely affect health and well-being as they wait in shelters for rental assistance that may not ever materialize. Furthermore, these efforts highlight that in prioritizing those with the greatest need, these systems may actually participate in creating need itself, by, for example, requiring individuals to reach a point of desperation before obtaining housing.

Some participants also described leveraging health needs to obtain priority for housing. In doing so, a few participated in health-related programs that they did not find helpful but considered a route to housing. Their efforts in this regard highlight the medicalization of housing (and poverty) that has been articulated elsewhere in the literature (Hansen, Bourgois, and Drucker 2014). As Hansen and colleagues describe in their ethnographic study of disability applications, illness and disability benefits have become survival strategies for individuals who lack alternative sources of income. Similarly, prior research on the intersections of housing and HIV suggests that positive HIV status or declining health was sometimes viewed as a way to access otherwise unavailable housing resources (Crane, Quirk, and Straten 2002).

Relatedly, participants’ experiences also reflect a system in which housing is used to *treat* illness, rather than prevent it. Indeed, some expressed the sentiment that the only way to obtain housing resources was to prove that their lack of housing had dire consequences for their health. Their perspectives echo scholars who have argued that investments in housing for poor communities are motivated by efforts to alleviate other costs to the State such as health care expenses (Fleming et al. 2019). Indeed, much of the policy discourse on housing and health has focused on supportive housing resources that are targeted to the small percentage of patients who account for a large percentage of health care spending (Cassidy 2016; Fleming et al. 2019). In contrast, there is less discussion of housing as a resource to prevent poor health or as a human rights issue. In such a framework, the simple lack of housing would be sufficient to indicate the need for it, rather than also requiring applicants to prove the extent or the consequences of this need.

Finally, our data suggest that the labor, uncertainty, stress, and surveillance associated with the application process may adversely affect the health and well-being of individuals who are waiting for rental assistance, potentially exacerbating any health consequences associated with their unmet housing needs (Fenelon et al. 2017; Keene et al. 2020). Indeed, the uncertain application process seemed to leave many participants feeling as if they had little control over their housing prospects, likely contributing to health demoting psychological stress. Furthermore, participants’ persistent efforts to obtain rental assistance may diminish their already limited time and emotional bandwidth, thus reducing their ability to attend to other health-related needs (Keene et al. 2018).

Though our findings provide an important perspective into the experiences of those seeking rental assistance, our study has some limitations. First, it can only speak to the perceptions of those seeking rental assistance, which may differ from the perspectives of policy administrators or the actual policies themselves. Nonetheless, these perceptions are important determinants of participants’ actions and critical to evaluating policy implementation and effectiveness. Additionally, our study addresses the experiences of individuals in one small city. Some aspects of our findings may not be transferable to other settings. For example, while most housing authorities have long waiting lists (the national average is two years), the challenges our participants describe may be very different in areas that do not have waiting lists. Additionally, the challenges that our participants describe with systems of prioritization may be very different in areas where housing authorities allocate housing through a lottery or on a first-come, first-served basis. Ultimately, our understanding of the rental application process can be further developed through additional in-depth qualitative studies in other locations and by comparing experiences across different policy contexts. Furthermore, our study was designed to examine a range of factors including rental assistance and did not sample explicitly on rental assistance status. Though we were able to capture participants’ experiences at varying points of the rental assistance application process, future research that selects and follows participants throughout the waitlist process is likely to provide additional important insights. Finally, a significant portion (one-third) of our sample never applied for rental assistance, despite income eligibility. More research is needed to explore potential barriers in the initial application process.

Despite these limitations and the need for further research, our study suggests some directions for policy and practice. In particular, it raises concerns about the utility of policies that allocate rental assistance through complex processes that depend on significant amounts of applicant knowledge, labor, and advocacy. As Moore (2016: 482) notes, any time the application process requires action from a participant there is the possibility that they “will be rationed out due to non-compliance with this activity.” For vulnerable populations, including those who are homeless or have a criminal justice history, the possibility of non-compliance and therefore failure to obtain assistance, may be disproportionately large. Furthermore, our findings highlight the challenges of assigning priorities for housing in a landscape where the need for this resource far outstrips the availability. In this respect, our findings, speak to a need for more housing resources and for policies that guarantee access to rental assistance for all eligible households.
